# Norovirus Extraction from Frozen Raspberries Using Magnetic Silica Beads

**DOI:** 10.1007/s12560-021-09466-0

**Published:** 2021-03-02

**Authors:** Philippe Raymond, Sylvianne Paul, André Perron, Louise Deschênes

**Affiliations:** 1 Canadian Food Inspection Laboratory (CFIA), St. Hyacinthe Laboratory, Food Virology, Saint Hyacinthe, QC Canada; 2 Agriculture and Agri-Food Canada (AAFC), St. Hyacinthe Research and Development Centre, Saint Hyacinthe, QC Canada

**Keywords:** Norovirus, Raspberries, RNA extraction, Silica, RT-qPCR

## Abstract

Human noroviruses (HuNoV) are among the main causes of acute gastroenteritis worldwide. Frozen raspberries have been linked to several HuNoV food-related outbreaks. However, the extraction of HuNoV RNA from frozen raspberries remains challenging. Recovery yields are low, and real-time quantitative reverse transcriptase PCR (RT-qPCR) inhibitors limit the sensitivity of the detection methodologies. A new approach using fine magnetic silica beads was developed for the extraction of HuNoV spiked on frozen raspberries. Relatively low recovery yields were observed with both the magnetic silica bead and the reference ISO 15216-1:2017 methods. High RT-qPCR inhibition was observed with the ISO 15216-1:2017 recommended amplification kit but could be reduced by using an alternative kit. Reducing RT-qPCR inhibition is important to limit the number of inconclusive HuNoV assays thus increasing the capacity to assess the HuNoV prevalence in frozen raspberries.

**Supplementary Information:**

The online version contains supplementary material available at 10.1007/s12560-021-09466-0.

## Introduction

Human Norovirus (HuNoV) is one of the leading causes of food-related illnesses in developed countries. HuNoV represents 58% of all reported foodborne outbreaks of known etiology in the United States (Vinje 2015). About 300–400 outbreaks of HuNoV are reported to the National Enteric Surveillance Program of the Public Health Agency of Canada each year (Government of Canada 2018). Forty-six berry outbreaks associated to HuNoV contamination with 15,827 cases were reported globally between 1983 and 2018 (Bozkurt et al. 2020). In the European Union, contaminated frozen red fruits were shown to represent an important cause of HuNoV outbreaks (Boqvist et al. 2018). Contaminated frozen raspberries are the most common sources (Bozkurt et al. 2020). Frozen raspberries were implicated as food vehicles in 33 of the 40 reported outbreak events associated with contaminated frozen product between 2008 and 2018 (Nasheri et al. 2019).

Noroviruses are small (27–40 nm) non-enveloped single-stranded RNA viruses that are transmitted mainly via the fecal–oral route. Noroviruses belong to a genetically diverse group of viruses of the *Caliciviridae* family. There are 10 distinct norovirus genetic groups (Chhabra et al. 2019). HuNoV GI and GII are the most prevalent genogroups associated with outbreaks. Noroviruses can persist in an infectious state for prolonged periods of time in the environment, in water, and in food (reviewed in Cook et al. (2016)). They can also withstand a broad pH range (pH 2–9). In cold temperatures, noroviruses can stay infectious for years. However, they are inactivated by cooking. The greatest risk of a foodborne HuNoV infection arises from the consumption of contaminated food such as fresh or frozen fruits, leafy vegetables, oysters, and drinking water.

Detection of HuNoV relies on the extraction of RNA and real-time reverse transcriptase PCR amplification methodologies (Vinje 2015). Prior to detection, minute amounts of viruses must be extracted from food matrices. Methods for concentrating noroviruses extracted from contaminated food are based mainly on ultrafiltration, ultracentrifugation, cationic separation, and polyethylene glycol (PEG) precipitation. These methods are associated with high variability and relatively low recovery of HuNoV (Summa and Maunula 2018). The ISO/TS 15216-1:2013 technical specification and the subsequent ISO 15216-1:2017 standard were published to provide a reference method for the quantitative extraction and detection of HuNoV from various food products. The extraction from soft fruit, leaf, stem, and bulb vegetables is based on PEG precipitation of the virus. However, PCR inhibitors are frequently reported when PEG-based approaches are applied to soft fruits and can lead to false-negative results (Summa and Maunula 2018). PCR inhibitors are also reported when using ultrafiltration, Cat-Floc precipitation, and immunomagnetic bead-based extraction methods as well, reducing the norovirus recovery yields substantially (Summa et al. 2012). Extraction methods with low recovery yields increase the probability of false-negative results. Assays with more than 75% real-time quantitative reverse transcriptase PCR (RT-qPCR) inhibition should be considered inconclusive (ISO 2017).

Other groups have used cationic beads or filters at high pH for norovirus adsorption and elution and have had various success rates (Morales-Rayas et al. 2010; Scherer et al. 2010; Stals et al. 2012). The recombinant HuNoV capsid protein VPI isoelectric point was reported to be pH 5 (Da Silva et al. 2011). Accordingly, norovirus should be positively charged below its capsid isoelectric point. Relatively high recovery yields (68%) for HuNoV GII from wastewater were reported using celite, a siliceous rock powder, at pH 4 (Brinkman et al. 2013). Silica surfaces are covered by silanol groups which can exist in different states. From previous studies, it is expected that crystalline and vitreous silica surfaces should maintain a negative charge above pH 2 and 3, respectively (Júnior and Baldo 2014). Andrade et al. (2009) reported an isoelectric point of 2.3 for silica-coated magnetic beads in suspension in KCl 1 mM.

In this study, we present the performance of a new magnetic silica bead (MSB) methodology for the extraction of norovirus. We compared the performance of this approach to the ISO 15216-1:2017 method with a focus on the RT-qPCR inhibition associated to the extraction and detection. Reducing RT-qPCR inhibition is important to limit the number of inconclusive assays.

## Methods

### Virus Stocks

Murine norovirus-1 (MNV) was provided by Dr. H. Virgin from Washington University (St. Louis, MO, USA). MNV was propagated in the RAW 264.7 cell line as previously described and viral stocks were titrated by plaque assays (Gonzalez-Hernandez et al. 2012). HuNoV-positive stool samples HuNoV GI.5 (CFIA-FVR-022) and GII.4 (CFIA-FVR-019) were provided by the British Columbia Center for Disease Control (BCCDC). The preparation of HuNoV from clarified 10% stool samples was adapted from Houde et al. (2006). Briefly, the stool samples were diluted in 1× phosphate-buffered saline (PBS), pH 7.4 (Gibco, Canada) to obtain a 10% suspension and were homogenized by vigorous agitation. Suspensions were clarified by centrifugation (20,000×*g*, 15 min, 4 °C) and kept frozen at − 80 °C.

### Frozen Raspberry Samples

Frozen raspberries, from bags labeled as whole individually quick frozen (IQF) collected at local stores, were subdivided in 25 g samples and used for artificial contamination experiments.

### Artificial Contamination of Frozen Raspberries

Aliquots of clarified 10% stools of HuNoV were vortexed for 2 s and diluted in PBS to the genomic equivalent copy (gEq) level needed in 100 µl per sample. To estimate the recovery yields, between 10^3^ and 10^6^ gEq of HuNoV GI.5, GII.4, and MNV were inoculated. To estimate the recovery yields in the presence of a competitor strain, 10^5^ gEq HuNoV GII.4 and 10^5^ gEq GI.5 were inoculated simultaneously. To estimate the limit of detection (LOD), the HuNoV GII.4 stocks were serially diluted to 10^5^–10^2^ gEq per 100 µl.

Frozen raspberries were spiked with the diluted viral suspensions on the surface of the food matrices (25 g) in a Whirl–Pak^®^ filter bag (VWR, Canada), then left to air dry 30 min in a biosafety cabinet. A frozen raspberries sample, with no virus added, was included in each extraction batch as a negative control. The amount of virus in the 100 µl inoculum was assessed in parallel by extracting the total RNA using the RNeasy kit (QIAGEN) followed by a RT-qPCR assay as described below.

### Virus Elution and RNA Extraction

**Fig. 1:**
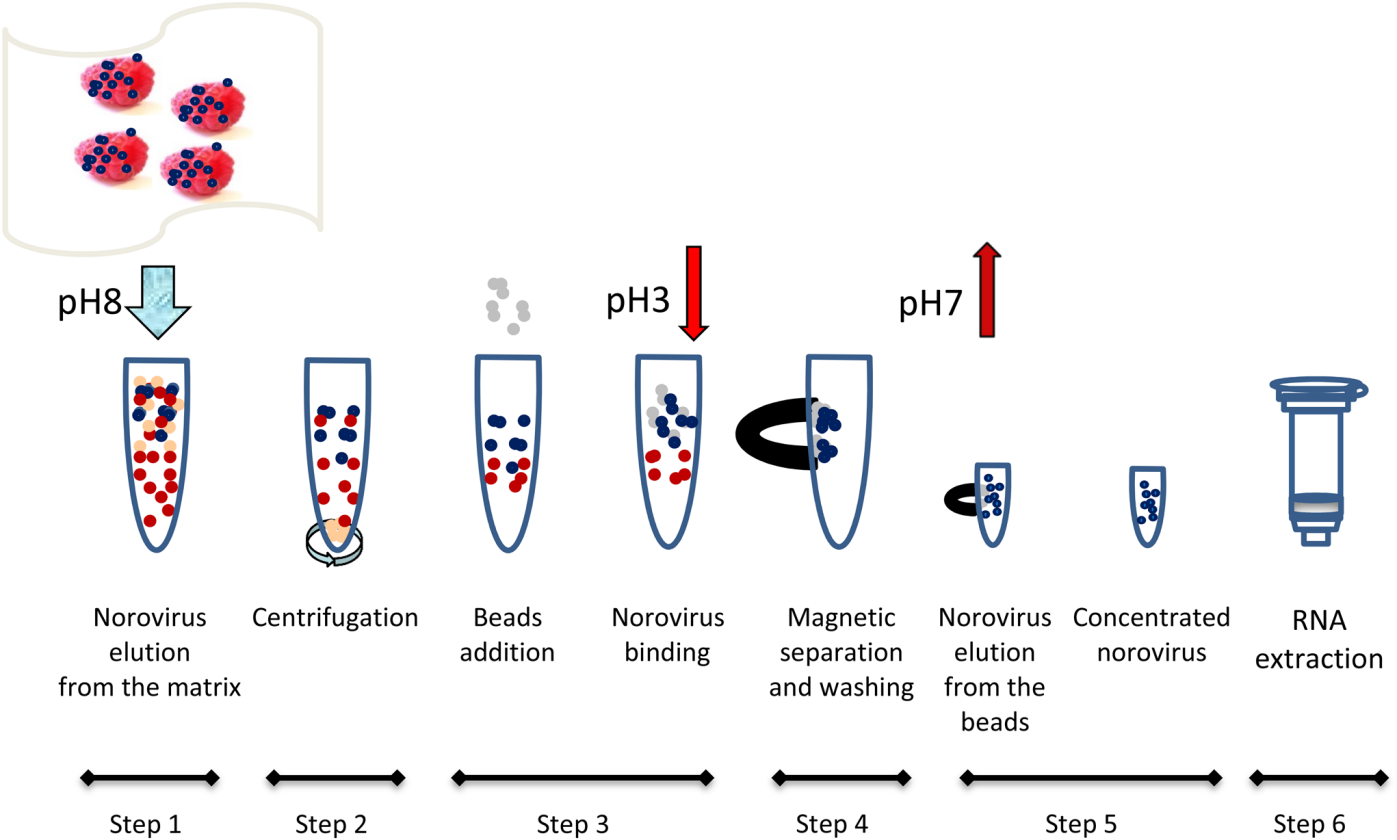
Norovirus extraction and concentration method using the magnetic silica beads (MSB) approach. Viruses (blue dots) are purified from matrix and soil contaminants (brown and red dots) using magnetic silica beads (gray dots) prior to the RNA extraction

Viral RNA was extracted from frozen raspberries using the MSB method by proceeding with the following steps (Fig. 1):

*Step 1* The virus was eluted from the matrix by adding 40 ml of elution buffer made of 150 mM Bis–Tris-Propane (Sigma-Aldrich, Canada) pH 8 to the sample in the filter bag then closing the bag and shaking it at about 55 RPM for 30 min at room temperature (RT) using an orbital shaker. Using a 25 ml borosilicate glass pipet, the eluate was transferred to a 50-ml conical centrifuges tubes.

*Step 2* The eluate was clarified by centrifugation at 3500×*g* for 10 min. Thirty units of *Aspergillus niger* pectinase (Sigma-Aldrich) were added to the clarified eluate followed by a 30 min incubation at 37 °C with shaking at approximately 70 RPM. Meanwhile, a volume of 100 µl per sample of AccuNanobeads (Bioneer, CA, USA, average size range 300–700 nm) was vortexed for 3 min. The AccuNanobeads storage buffer was removed using a magnet, and the beads were resuspended in 1 ml per sample of the elution buffer containing 100 µg/ml Pluronic F-127 (Anatrace, OH, USA) and vortexed for a few seconds.

*Step 3* The bead suspension was added to the clarified eluate and vortexed 3 s. Two milliliters of 200 mM ascorbic acid (Sigma-Aldrich) and 3.5 ml of 200 mM malic acid (Sigma-Aldrich) were added to each sample, and the tubes were vortexed another 3 s. The pH was lowered between pH 2 and 3 with the addition of approximately 850 µl of 6 N HCl. The tubes were then mixed using a Dynal rotary shaker 94701 (Thermo Fisher, Canada) set between 19 and 22, for 10 to 60 min at RT.

*Step 4* The solution was separated from the beads using a magnetic rack and removed by decantation. The remaining beads were washed with 1 ml of washing buffer composed of 17.4 mM Bis–Tris-Propane buffer pH 7 with 8.7 mM NaCl, 0.87 mM CaCl_2_, 10.9 mM ascorbic acid, and 15.2 mM malic acid.

*Step 5* The viruses were eluted twice from the beads with 100 µl of the bead elution buffer composed of 45 mM Bis–Tris-propane pH 9, 0.01% Tween 20, and 50 mM EDTA (Sigma-Aldrich). Beads were vortexed 3 s with the elution buffer. The first and second elutions were performed by agitation at about 1050 RPM in a Thermomixer R (Eppendorf, Canada) at RT for 10 min and 1 min, respectively. Virus eluates were immediately transferred to a 1.5 ml microtube and combined with 500 µl buffer RLT plus β-mercaptoethanol from the RNeasy QIAcube kit (QIAGEN) or 10 µl of 2 M Dithiothreitol (Sigma-Aldrich). Five microliters of RNA carrier (1 µg/ml) (QIAGEN) and 140 µl of the polyvinylpolypyrrolidone (PVPP; Sigma-Aldrich) suspension (2% v/v final) were added to each sample, and the samples were vortexed briefly. Samples were centrifuged at 10,000×*g* minimum for 5 min to remove the PVPP.

*Step 6* Total RNA was extracted using the RNeasy QIAcube kit supplemented with DNase I in the QIAcube platform as described by the manufacturer (QIAGEN). The RNA was eluted from the spin column membrane with 50 µl of RNase-free water from the kit to which 40 units of the RNasin Plus RNase inhibitor (Fisher Scientific, Canada) were added. It was then stored at − 80 °C.

As a reference method, the ISO 15216-1:2017 method for soft fruit samples (ISO 2017) was also used for the extraction of the viruses from spiked frozen raspberries. The pectinase from *Aspergillus aculeatus (1140 U)* was used. The NucliSens miniMAG kit (Biomérieux, Canada) was used to extract RNA in 50 µl elution buffer following the manufacturer’s recommendations.

### RT-qPCR

RT-qPCR assays were performed using 5 µl of diluted (1/10) or non-diluted RNA extracts, using either the Mx3005P system (Stratagene, CA, USA) or the Quantstudio 6 system (Thermo Fisher). Diluted RNA extracts were prepared using RNase-free water. Unless otherwise specified, HuNoV GII RT-qPCR was performed using QNIF2d and COG2R primers and the probe QNIFS (Table1) following the procedure described in ISO 15216-1:2017 (ISO 2017). HuNoV GI RT-qPCR was performed using QNIF4 and NV1LCR primers (Da Silva et al. 2007; Svraka et al. 2007) with the TM9 probes (Hoehne and Schreier 2006). MNV detection was performed based on RT-qPCR ORF1/ORF2 primer system developed by Baert et al. (2008). In both cases, 5 µl from the RNA extracts were tested using the TaqMan Fast Virus 1-Step Master Mix (Thermo Fisher). The reverse transcription was performed at 50 °C for 20 min, and the amplification profile included 20 s at 95 °C, and 45 cycles of 3 s at 95 °C and 30 s at 60 °C.

**Table 1:** Primers, probes, and RNA transcripts used in this study

Methods	Primer or probe	Sequence 5′–3′	References
Norovirus GI			
RT-qPCR, qPCR	QNIF4	CGC TGG ATG CGN TTC CAT	Da Silva et al. (2007)
RT-qPCR, qPCR	NV1LCR	CCT TAG ACG CCA TCA TCA TTT AC	Svraka et al. (2007)
RT-qPCR, qPCR	FAM-TM9-MGBNFQ	TGG ACA GGA GAT CGC	Hoehne and Schreier (2006)
PCR	GISKR	CTG CCC GAA TTY GTA AAT GA	Kojima et al. (2002)
PCR	GISKF	CCA ACC CAR CCA TTR TAC A	Kojima et al. (2002)
Norovirus GII			
RT-qPCR, qPCR	QNIF2d	ATG TTC AGR TGG ATG AGR TTC TCW GA	Loisy et al. (2005)
RT-qPCR, qPCR	FAM-QNIFS-BHQ-1	AGC ACG TGG GAG GGC GAT CG	Loisy et al. (2005)
RT-qPCR, qPCR	COG2R	TCG ACG CCA TCT TCA TTC ACA	Kageyama et al. (2003)
Murine norovirus			
RT-qPCR	FW-ORF1/ORF2	CAC GCC ACC GAT CTG TTC TG	Baert et al. (2008)
RT-qPCR	RV-ORF1/ORF2	GCG CTG CGC CAT CAC TC	Baert et al. (2008)
RT-qPCR	FAM-ORF1/ORF2-MGBNFQ	CGC TTT GGA ACA ATG	Baert et al. (2008)

Virus gEq quantification was determined using standard curves generated with in vitro RNA transcripts containing target sequences for HuNoV GI, GII, or MNV with small sequence inserts to differentiate them from the circulating strains. The HuNoV GII RNA transcript UV concentration was divided by a correction factor of 1.8, established by droplet digital PCR (ddPCR) (Advance Analysis Centre Guelph University, Canada). Briefly, reverse transcriptase reactions were performed in triplicate as described above using the COG2R primer. The Automated Droplet Generator (Bio-Rad, Canada) was used to generate droplets. QPCRs were performed as described below using the C1000 touch Thermal cycler and ddPCR Multiple Supermix (Bio-Rad). Plates were read using the QX200 Droplet Reader (Bio-Rad).

### Recovery Yield Calculation

The recovery yields associated with the virus elution and concentration steps were estimated using the cycle thresholds (Ct) variation. The virus recovery yields = 10^(Δ*C*t/*m*)^ × 100%, where Δ*C*_*t*_ = *Ct*_matrix_* − Ct*_inoculum_ is the *Ct*_matrix_ value of extracted viral RNA from the matrix minus the *Ct*_inoculum_ value of viral RNA extracted from the inoculum, and *m* is the slope of the virus RNA transcript standard curve.

For the MSB method, the inoculum viral RNA levels were estimated from the extraction of 100 µl of the virus diluted with 100 µl of the bead elution buffer using the RNeasy Qiacube kit and its analysis by RT-qPCR.

For the ISO 15216-1:2017 method, the inoculum viral RNA levels were estimated from the extraction of 100 µl of the virus dilution using the NucliSens miniMAG kit and its analysis by RT-qPCR.

### RT-qPCR Inhibition

The RT-qPCR inhibition from the matrix was evaluated as recommended in ISO 15216-1:2017 using RNA transcripts with insert as an external amplification control (EAC). Briefly, non-spiked raspberry samples were extracted using the MSB or the ISO 15216-1:2017 protocol as described above. Five microliter of RNA extract was spiked with 625 gEq of EAC and tested by RT-qPCR using either the RNA UltraSense or the TaqMan Fast Virus 1-Step Master Mix kit as described above. EAC spiked in RNase-free water was used as controls.

RT-qPCR inhibition rate = (1 − 10^(Δ*Ct/m*)^) × 100% where Δ*C*_*t*_ = *Ct*_matrix_ − *Ct*_water_ is the *Ct*_matrix_ value of RNA transcript spiked in RNA extracted from the matrix minus *Ct*_water_ value of RNA transcript in water and *m* is the slope of the virus transcript RNA standard curve.

### Limit of Detection (LOD) Calculation

The PODLOD program (v9) (Wilrich and Wilrich 2009) was used to calculate the LOD_50_ and LOD_95_.

### Statistical Analyses

Unless otherwise specified, all statistical analyses were performed on log-transformed values using the independent samples *t* test (*p* > 0.05). The F-test was performed to evaluate the variance. A two-way analysis of variance (ANOVA) with the Bonferroni statistical correction was used to evaluate the impact of the extraction and detection method on the RT-qPCR inhibition (*p* > 0.05) (MedCalc 17.5.5).

## Results

### Recovery Yields

The performance of the MSB method was evaluated by assessing its recovery yields in comparison to the ISO 15216-1:2017 method using IQF frozen raspberries spiked with HuNoV GII.4, GI.5, or MNV (Table 2). In our laboratory setting, the virus elution and concentration methods for norovirus using the MSB had a turnaround time of approximately 7 h, including 1 h required for the robotic RNA extraction. The ISO 15216-1:2017 method had a turnaround time of 9 h, including the inoculation and the manual NucliSens miniMAG RNA extraction performed on the second day. We had some difficulty resuspending the pellet after the PEG precipitation step when 30 units of pectinase from *A. niger* were used as indicated in the ISO/TS 15216-1:2013 (data not shown). When the pectinase from *A. aculeatus* was used as indicated as an alternative in the ISO 15216-1:2017 protocol version, the pellet was resuspended more easily. Nevertheless, the HuNoV GII.4 recovery yields from frozen raspberries using the MSB and ISO 15216-1:2017 methods were not statistically different (*p* = 0.366) and were 2.6% and 1.8%, respectively. When high (5 × 10^4^ gEq) or low amounts of viruses (1.7 × 10^3^ gEq) were spiked and extracted using the MSB method, the HuNoV GII.4 recovery yields were again not statistically different (*p* = 0.855). Recovery yields for HuNoV GI.5 were similar to HuNoV GII and calculated to be 3.6%. Repeated freezing and thawing of raspberry matrices before the virus elution appeared to release more pectin and/or the formation of agglomerates that precluded the magnetic beads handling. Consequently, frozen raspberry matrices were classified as unfit if they had been received thawed and were not used to evaluate recovery yields.

**Table 2:** Detection of norovirus in spiked frozen raspberries

Extraction method	Virus	Spiking level^a^	*n*	Undiluted		Diluted (1/10)	
				Ct^b^	Recovery yields^c^	Ct^b^	Recovery yields^c^
MSB	MNV	2.4 × 10^6^	15	26.0 ± 0.6	2.8% (1.8–3.8)	nt	nt
	GII.4	5 × 10^4^	15	29.7 ± 1.2	2.6% (1.7–5.0)	30.4 ± 2.5	6.2% (4.2–8.2)
	GII.4	1.7 × 10^3^	5	34.0 ± 1.5	5.7% (0.8–6.6)	nt	nt
	GI.5	6 × 10^4^	15	29.2 ± 0.8	3.6% (2.4–4.9)	32.8 ± 0.8	3.0% (2.1–3.9)
15216	GII.4	5 × 10^4^	15	29.8 ± 1.9	1.8% (0.1–3.6)	30.8 ± 1.5	9.3% (7.1–11.5)

*n* extraction assay, *nt* not tested, *Ct* cycle threshold, *MSB* magnetic silica beads, *15216* ISO 15216–1:2017

^a^Genomic equivalent copies

^b^Average Ct ± standard deviation

^c^Geometric mean (95% confidence interval)

As for MNV, the recovery yield from spiked frozen raspberries using the MSB method was 2.8%, similar to the HuNoV GI and GII recovery yields (One-way ANOVA, *p* = 0.431). The tissue culture infectious dose (TCID_50_) of the MNV production batch was titrated at 203 gEq/TCID_50_ (CI 95% 139–297; *n* = 7). With a recovery yield of 2.8% using MSB, it was extrapolated that 25 g of frozen raspberries spiked with 100 µl of a MNV at 53 TCID_50_/ml should allow 3 MNV genomic copies in the RT-qPCR tubes to be detected 94% of the time according to the Poisson distribution.

Moreover, the addition of a competitor strain did not have any impact on norovirus recovery yields from spiked frozen raspberries using the MSB method. Indeed, the HuNoV GII recovery yields in the presence or absence of HuNov GI.5 were in the same range 3.4% and 4.5%, respectively (*p* = 0.247) (Table 3).

**Table 3:** Norovirus recovery yields from frozen raspberries in the presence of competition

Extraction method	*n*	Virus			Competitor virus		
		Virus	Spiking level^a^	Recovery yields^b^	Virus	Spiking level^a^	Recovery yields^b^
MSB	5	GII.4	3 × 10^5^	4.5% (3.5–5.8)	GI.5	10^5^	3.0% (2.0 to 5.0)
	5	GII.4	4 × 10^4^	3.4% (1.8–6.2)	nt	nt	nt
	5	nt	nt	nt	GI.5	10^5^	1.8% (− 0.4 to 4.0)

Raspberries spiked with or without competitor virus were extracted and tested by RT-qPCR

*n* extraction assay, *nt* not tested, *MSB* magnetic silica beads

^a^Genomic equivalent copies

^b^Geometric mean (95% confidence interval)

### RT-qPCR Inhibition

The ratio of diluted (1/10) to non-diluted HuNoV GII RNA recovery yields indicated a fairly high relative RT-qPCR inhibition value of 5.2 when the HuNoV GII RNA was extracted from frozen raspberries using the ISO 15216-1:2017 method compared to a ratio value of 2.4 observed with the MSB method (Table 2). Ratio values above 4 represent more than 75% RT-qPCR inhibition. In contrast, the tenfold dilution of the HuNoV GI RNA from samples extracted using the MSB method on frozen raspberries had no impact on the recovery yields (*p* = 0.214). The average undiluted HuNoV GI RNA extract Ct was not lower than the diluted ones after correction for the dilution factor.

**Fig. 2:**
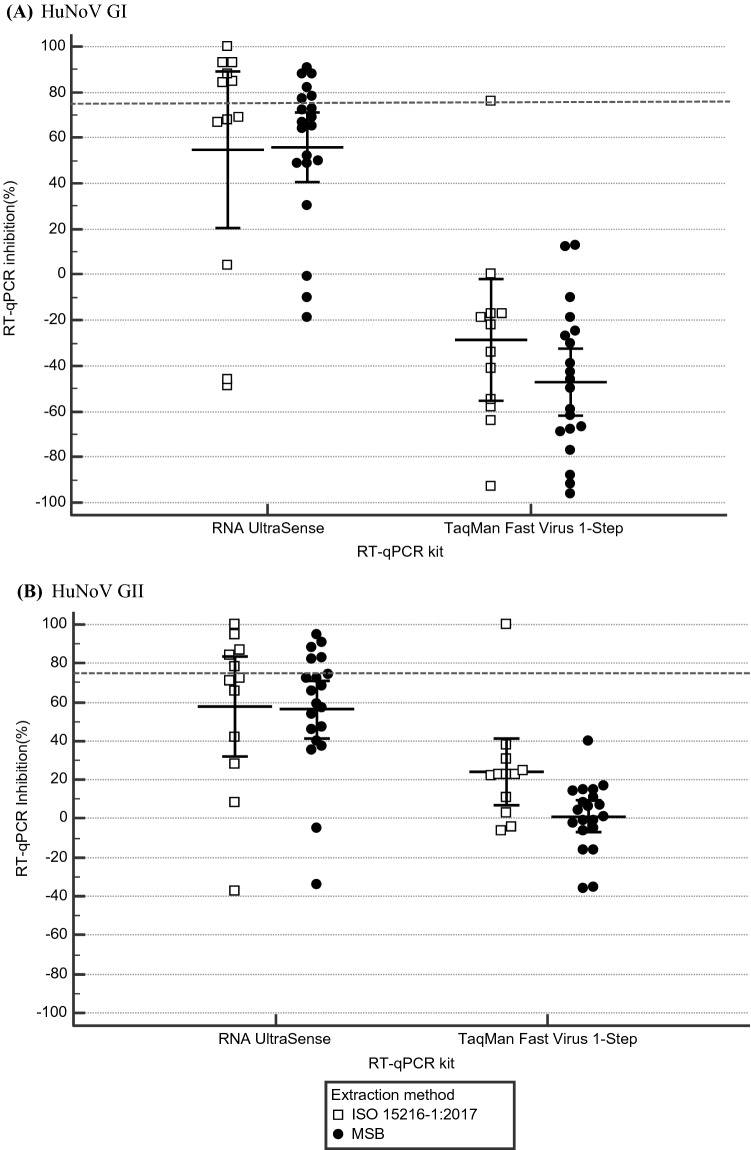
RT-qPCR inhibition percentages in frozen raspberry RNA extracts. A box plot of RT-qPCR inhibition evaluated using **a** HuNoV GI and **b** HuNoV GII external control RNA amplification is shown. The absence of RT-qPCR inhibition or enhancement should read as 0. The error bars represent the 95% confidence intervals of the mean. The horizontal dotted line represents 75% inhibition

Using the HuNoV GII and GI RNA transcripts with insert as EAC, a statistically significant impact of the RT-qPCR kit (*p* < 0.001) on the RT-qPCR inhibition was observed (Fig. 2). The average RNA UltraSense HuNoV GII RT-qPCR inhibition percentages measured using the RNA extracted with the ISO 15216-1:2017 and the MSB approaches were 58% (95% CI 32–83) (*n* = 12 replicates) and 56% (95% CI 41–71) (*n* = 20), respectively. However, when testing the HuNoV GII EAC amplification using the TaqMan^®^ Fast Virus 1-Step, the average RT-qPCR inhibition percentages calculated with the RNA extracts using the ISO 15216-1:2017, and the MSB protocols were decreased to 24% (95% CI 7–42) (*n* = 12) and 1% (95% CI 7 to 9) (*n* = 20), respectively. Similar EAC inhibition levels were observed using the HuNoV GI RT-qPCR. RT-qPCR inhibition values were above the 75% when tested with the RNA UltraSense kit for 46% and 25% of the RNA samples extracted with the ISO 15216-1:2017 and the MSB methods, respectively. There was no impact of the extraction method on the RT-qPCR inhibition (*p* = 0.110).

### Limit of Detection

The limit of detection of the MSB method was evaluated by RT-qPCR using RNA extracted from frozen raspberries spiked with HuNoV GII.4 strain CFIA-FVR-019 (Supplementary Fig. SF1). Overall, 70 spiked and 14 non-spiked frozen raspberry samples were tested. The MSB RT-qPCR LOD_95_ and LOD_50_ were 2370 gEq per 25 g (95% CI 1542–3642) and 548 gEq per 25 g (95% CI 357–843), respectively.

## Discussion

Frozen raspberries have been associated with several norovirus outbreaks and remain a challenging food matrix for virus detection. We have experienced some difficulties with the workflow of the ISO/TS 15216-1:2013. The limited pH range during elution, the impact of PCR inhibitors, and a tedious PEG pellet resuspension observed with frozen raspberry matrices make this method poorly adapted to testing the large number of samples CFIA diagnostic labs need to process every year (> 500). Some of these issues were resolved in the 2017 version of the method. As an alternative, we developed a new food virus extraction method based on magnetic silica beads (MSB) which was better adapted to our laboratory setting. The MSB approach to elute and concentrate norovirus from raspberries is based on a strategic use of electrostatic interactions at different steps of the protocol. The variations of the virus surface charge at different pH levels are illustrated in Fig. 1. According to the manufacturer, the fine magnetic silica beads have a small size (420 nm) and have a negative zeta potential in water (− 37 mV). On the other hand, silica particles are prone to aggregation in the presence of salts (Metin et al. 2011). In the MSB method, a non-ionic buffer (Bis–Tris-Propane) was used, and salts were avoided in the buffer solutions to reduce the ionic strength and to limit the aggregation of magnetic silica particles. The effect of pH and charge of the norovirus were also taken into account. According to Da Silva et al. (2011), HuNoV GI Virus-like particles (VLP) are prone to adhesion onto silica below the VLP isoelectric point whereas its attachment is reduced at higher pH at low salt concentrations.

It is difficult to compare method performance based on the literature or laboratory reports (Li et al. 2018). In addition to the extraction protocols, variations in recovery yields could be associated to the inoculated virus preparation, clarification, filtration, the virus strain, its integrity, as well as the spiking conditions. The method should be fit for its intended purpose and detects HuNoV in frozen raspberries at level equivalent to the 50% human infectious dose (HID50). The HID50 of the HuNoV in susceptible healthy adults varies with the serogroup. The HID50 of the HuNoV Norwalk strain was estimated at 1320 (95% CI 440–3760) genomic equivalent (gEq) in serogroups O and A (Atmar et al. 2014). The HuNoV GII LOD_95_ reported in this study using the MSB approach was higher (2 × 10^3^ vs. 0.7 × 10^3^ gEq per 25 g) than the ones reported by another study using the ISO 15216-1:2017 (Li et al. 2018). On the other hand, recovery yields obtained for undiluted HuNoV GI and HuNoV GII using the MSB method were in the same range (2.6–5.7%) as the ISO 15216-1:2017 reported by Fraisse et al. (2017). While achieving the lowest detection limit is a major goal of extraction methodologies, its application also influences method selection. For instance, methodologies that could discriminate between inactivated and infectious virus are required to avoid overestimating the viral infectivity. Viability RT-qPCR based on viral integrity treatment is a promising approach to improve risk assessment of positive RT-qPCR detection results (Chen et al. 2020). The integrity of the virus following its elution and concentration might varied between different viral RNA extraction methodologies and requires further investigation.

Raspberries contain high levels of heteropolysaccharides, such as pectin, which have a major impact on the viral extraction process. The pectin appears to form a strong gel with calcium at a pH close to 5 which interferes with the elution process (Han et al. 2017). Pectinase treatment, lower pH, and low calcium conditions were required to avoid bead agglomeration when performing the MSB elution from frozen raspberries. The current low pH extraction process was effective with a limited set of matrices.

In addition to pectin, the extraction of HuNoV from frozen raspberries presented other challenges. Raspberries contain multiple components that are co-extracted with the viral genome and can impact its molecular detection. High levels of polyphenols (e.g., anthocyanin, flavonol, ellagitannin, proanthocyanidin, phenolic acids, tannic acid) can act as PCR inhibitors (reviewed in Schrader et al. (2012)). Heteropolysaccharides can disturb the RT and PCR enzymatic process by mimicking the structure of nucleic acids. Phenolic compounds may cross‐link RNA under oxidizing conditions and could degrade DNA polymerases. To reduce the presence of PCR inhibitors, the MSB method includes a treatment with insoluble polyvinylpolypyrrolidone that was reported to prevent polyphenol oxidation and subsequent binding to nucleic acids when extracting total RNA from raspberries (Jones et al. 1997).

High levels of RT-qPCR inhibition have been reported by several groups that evaluated PEG-derived extraction methods including ISO/TS 15216-1:2013 and ISO 15216-1:2017 with frozen raspberries (De Keuckelaere et al. 2015; Fraisse et al. 2017; Summa and Maunula 2018; Summa et al. 2012). With frozen raspberries RNA extracted using the ISO 15216-1:2017, RT-qPCR inhibition was estimated at 93.8% ± 2.5% using an external RNA control (Fraisse et al. 2017). According to the ISO 15216-1:2017, negative results obtained in presence of RT-qPCR inhibition levels > 75% are not valid.

Meanwhile in this study, close to 50% of the undiluted samples extracted following the ISO 15216-1:2017 protocol and tested using the RNA UltraSense detection kit presented more than 75% inhibition. Such a level of inconclusive assays increases the burden of testing and could double reported prevalence estimates if this factor was not taken into account. The ISO 15216-1:2017 recommends a larger elution volume (100 µl vs 50 µl) and requires testing a tenfold diluted RNA extract as well as testing EAC to avoid this issue (ISO 2017). Previously, a European survey reported that positive frozen raspberries contaminated with HuNoV were only detected using tenfold diluted RNA when RNA was extracted with the ISO/TS 15216-1:2013 method (Loutreul et al. 2014). Dilution of the soft fruit RNA extract decreases the impact of PCR inhibitors in the RT-qPCR. However, RNA extract dilution also impacts the capacity to detect the virus present at trace levels (Fraisse et al. 2017). Until improvements are shown regarding the recovery yields, strategies that decrease the impact of PCR inhibitors from frozen raspberry RNA extracts should be encouraged.

Different PCR inhibitor removal kits or the use of digital PCR have improved the ISO 15216-1: 2017 recovery yields (Fraisse et al. 2017; Bartsch et al. 2016, 2018). For instance, an additional RNA purification step using either the MobiSpin column or the OneStep^®^ PCR Inhibitor Removal Kit (Zymo research) were added with the ISO/TS 15216-1:2013 protocol to remove RT-PCR inhibitors from frozen strawberries and improve the detection limit (Bartsch et al. 2016, 2018). Alternative extraction approaches could also reduce the amount of RT-PCR inhibitors. With Bovine Norovirus spiked on frozen raspberries, Sun et al. (2019) have reported the absence of RT-PCR inhibition using a direct lysis approach combined with either RNA filtration using the MobiSpin column or digital PCR detection. The results in this study indicated that the RT-qPCR inhibition is also influenced by the selected detection method. The RNA UltraSense kit is described in the ISO 15216-2017 method, but it is not a requirement for the method. The RNA UltraSense kit might be suitable for some food matrices included in the scope of this method that are associated to low RT-qPCR inhibition. However, the TaqMan Fast Virus 1-Step results indicate that the UltraSense kit is not the most appropriate detection kit to test RNA extracted from frozen raspberries. The impact of PCR inhibitors from frozen raspberry RNA extracts on other commercial RT-qPCR kit was not explored. Commercial RT-qPCR kits use different proprietary buffers and additives to reduce the impact of PCR inhibitors. The extracted PCR inhibitors vary with the type of food matrix. Consequently, the impact of inhibitors on commercial RT-qPCR kits should be tested for each type of food matrix.

Several groups have used various forms of the ISO 15216 method to recover HuNov from frozen raspberries. The observed range of recovery yields from 1 to 6% range could certainly impact the reported prevalence. Nevertheless, Loutreul et al. (2014) reported a prevalence of 16.7% (*n* = 162) for HuNov GI in frozen raspberries from Serbia, Chile, Bulgaria, Poland, and France. In the UK, 3.6% (*n* = 274) of frozen raspberry samples sold at retail were positive for HuNoV (Cook et al. 2019). However, this group was not able to differentiate the sequence of the HuNoV detected from their EAC. Gao et al. (2019) reported that 9.2% and 13% of frozen raspberries from Heilongjiang Province in China were positive to HuNoV in 2016 and 2017, respectively. Meanwhile, all export samples were negative. They used a replicate Ct threshold for positive results, which could lower prevalence estimate, and did not analyze the RT-qPCR inhibition level. Maunula et al. (2013) did not detect HuNov in frozen raspberry samples (0/39) from point of sale of four European countries, but did find some HuNov GII in irrigating water from berry production sites (2/56). They used high pectinase concentration and RNA extract elution volume (300 µl) for berries but did not report any RT-qPCR inhibition. The true prevalence as well as the extraction method recovery yields and the presence of PCR inhibitors could play a role in resolving some of the discrepancies observed in terms of prevalence between those groups. Still, the contamination levels in some of these surveys remains relatively important from a risk analysis perspective.

## Conclusion

A method based on magnetic silica beads to extract HuNoV virus RNA from frozen raspberries was developed. The MSB method performance was similar to the reference method ISO 15216-1:2017. The influence of RT-qPCR inhibitors extracted using both methods was reduced using an alternative RT-qPCR detection kit (TaqMan Fast Virus 1-Step Master Mix) and condition. In future, the reduction of the RT-qPCR inhibitors which impact the HuNoV detection results should reduce the number of inconclusive assays and influence prevalence estimates.

## Supplementary Information

Below is the link to the electronic supplementary material.

Supplementary Information 1 Supplementary Figure 1. Estimated probability of detection (POD) curve of HuNoV GII extracted from spiked frozen raspberries using the MSB extraction method and the RNA UltraSense RT-qPCR detection kit. Upper (U) and lower (L) POD 95% confidence bands are represented with dash and dot, respectively. Each observed value represents five extractions. (DOCX 18 KB)
