# Supplementary material for: Norovirus Extraction from Frozen Raspberries Using Magnetic Silica Beads
Source: Food Environ Virol. 2021 Mar 2;13(2):248–58. doi: 10.1007/s12560-021-09466-0 (PMC8116234; doi:10.1007/s12560-021-09466-0)
Supplement: Supplementary file 1 — Supplementary Information 1 Supplementary Figure 1. Estimated probability of detection (POD) curve of HuNoV GII extracted from spiked frozen raspberries using the MSB extraction method and the RNA UltraSense RT-qPCR detection kit. Upper (U) and lower (L) POD 95% confidence bands are represented with dash and dot, respectively. Each observed value represents five extractions. (DOCX 18 KB) [file 12560_2021_9466_MOESM1_ESM.docx]

**Electronic Supplementary Material**
